# Does evaluation of in vitro microfilarial motility reflect the resistance status of *Dirofilaria immitis* isolates to macrocyclic lactones?

**DOI:** 10.1186/s13071-017-2436-6

**Published:** 2017-11-09

**Authors:** Mary J. Maclean, Molly D. Savadelis, Ruby Coates, Michael T. Dzimianski, Corey Jones, Cynthia Benbow, Bobby E. Storey, Ray M. Kaplan, Andrew R. Moorhead, Adrian J. Wolstenholme

**Affiliations:** 1 Department of Infectious Diseases, University of Georgia, 501 DW Brooks Drive, Athens, 30602 GA USA; 2 Center for Tropical and Emerging Global Diseases, University of Georgia, Athens, GA 30602 USA; 3 Yazoo City Animal Hospital, Yazoo City, MS USA; 4 Benbow Veterinary Services, Metairie, LA USA

**Keywords:** Heartworm, Drug resistance, Macrocyclic lactones, Ivermectin, Microfilarial motility

## Abstract

**Background:**

Several reports have confirmed that macrocyclic lactone-resistant isolates of *Dirofilaria immitis* are circulating in the United States; however, the prevalence and potential impact of drug resistance is unknown. We wished to assess computer-aided measurements of motility as a method for rapidly assessing the resistance status of parasite isolates.

**Methods:**

Blood containing microfilariae (MF) from two clinical cases with a high suspicion of resistance was fed to mosquitoes and the resultant L3 injected into dogs that were then treated with six doses of Heartgard® Plus (ivermectin + pyrantel; Merial Limited) at 30-day intervals. In both cases patent heartworm infections resulted despite the preventive treatment. Microfilariae isolated from these dogs and other isolates of known resistance status were exposed to varying concentrations of ivermectin in vitro and their motility assessed 24 h later using computer-processed high-definition video imaging.

**Results:**

We produced two isolates, Yazoo-2013 and Metairie-2014, which established patent infections despite Heartgard® Plus treatments. Measurements of the motility of MF of these and other isolates (Missouri, MP3 and JYD-27) following exposure to varying concentrations of ivermectin did not distinguish between susceptible and resistant heartworm populations. There was some evidence that the method of MF isolation had an influence on the motility and drug susceptibility of the MF.

**Conclusions:**

We confirmed that drug-resistant heartworms are circulating in the southern United States, but that motility measurements in the presence of ivermectin are not a reliable method for their detection. This implies that the drug does not kill the microfilariae via paralysis.

## Background

*Dirofilaria immitis*, the canine heartworm, is one of the most pathogenic parasitic helminths infecting small companion animals [1]. Infection in domesticated dogs and wild canids is widespread in the United States and other parts of the world. In domestic dogs the disease is prevented by prophylactic administration of one of several macrocyclic lactone (ML) products approved for this indication. These drugs have been shown to be 100% effective against the establishment of patent infections and the occurrence of subsequent disease when given in accordance with the manufacturers’ instructions [2–4]. The MLs are the only class of drug currently used for heartworm prophylaxis. Reports suggestive of drug failures (loss of efficacy), however, have been accumulating for more than 10 years [5–7] and recently the existence of resistant isolates of *D. immitis* has been unequivocally confirmed by the establishment of patent infections under laboratory conditions despite the use of appropriate ML treatments [8–11]. Most reports of ML resistance in *D. immitis* have focused on the Mississippi Delta region of the southern United States, an area of very high parasite challenge, but we do not know the frequency and geographic range of resistant isolates, and so it is difficult to assess the potential impact they might have on canine and feline health [2, 11]. In large part this is because the confirmation of drug resistance in heartworms is time-consuming and expensive. The in vivo microfilarial suppression test proposed by Geary et al. [12] appears to be useful, but there is still a need for a properly validated in vitro test for resistance. Evans et al. used a larval migration inhibition assay to measure the susceptibility of *D. immitis* L3 to MLs [13], but the concentrations of drug needed to prevent migration were much higher than those to which the larvae would be exposed to in vivo and the assay does not seem to be able to distinguish resistant from susceptible isolates [14]. Bourguinat et al. [5, 6, 15] used observations of microfilaria (MF) motility in a single concentration of ivermectin, described as twice the IC_95_, to assess the susceptibility and resistance of some candidate ML-resistant isolates. The derivation of the IC_95_, however, was by extrapolation from a limited number of observations at lower drug concentrations and the assays were dependent on visual observation and assessment of motility by an expert technician, which would limit the wider usefulness of the method.

The MLs are most effective against the infecting L3/L4 stage of the parasite life cycle, with clearance of circulating MF and any effects on the adult worms requiring much higher drug concentrations [16]. Microfilariae are by far the most convenient stage of the life cycle to use in any resistance assay, however, because they can be easily obtained from infected animals using noninvasive methods.

Recently, we reported the application of computer-processed high-definition video imaging (the ‘Worminator’) to measuring the motility of MF from *D. immitis* and *Brugia malayi* [17]. In this study, we evaluated the suitability of such methods for assessing the drug resistance status of several *D. immitis* isolates of known resistance status. Such experiments required the isolation and characterization of some new resistant isolates, Yazoo-2013 and Metairie-2014, and a description of these isolates is provided in this article.

## Methods

### Isolation of Yazoo-2013 and Metaririe-2014

Male purpose-bred beagles were purchased from Covance Inc. (Princeton, NJ, USA) with no previous exposure to macrocyclic lactones. Dogs were infected by subcutaneous injection of *D. immitis* L3. Thirty days after inoculation of *D. immitis* L3, dogs were administered Heartgard® Plus (ivermectin + pyrantel; Merial Limited) according to manufacturer’s recommendations. A total of six monthly treatments of Heartgard® Plus were administered every 30 days. Microfilariae concentrations were determined using thick smears as described [18].

#### Yazoo-2013

Yazoo-2013 was isolated from a 2-year-old intact male Doberman pinscher that resided in Yazoo City, Mississippi, USA. Trifexis® (milbemycin oxime + spinosad; Elanco) was administered at a weight-appropriate dose beginning at 8 months of age. Per preventive heartworm treatment standards, this dog was heartworm negative when Trifexis® was first given in September 2011 and remained so for another test in July 2012. The owner purchase history verified that drug was purchased monthly through July 2013, when the dog tested antigen and MF positive. The owner was considered highly compliant and attentive to treatment by the attending veterinarian, and reported that the dog took the drug tablet willingly every month and did not vomit after administration. The same day the dog tested positive, Trifexis® was administered to prevent transmission before beginning adulticidal treatment, and blood was sent to the University of Georgia 30 days later for feeding to mosquitoes. This produced 100 L_3_, which were evenly divided and injected subcutaneously into two male purpose-bred beagles. Thirty days after infection with L_3_ each dog was administered Heartgard® Plus according to weight. Administration of Heartgard® Plus was repeated every 30 days for a total of 6 months after infection. One month after the final administration of Heartgard® Plus, blood was drawn to perform thick smears and an IDEXX SNAP® test to detect MF and heartworm antigen, respectively. Both dogs were MF and antigen positive 7 months after they were infected. Microfilaremia increased in both dogs for several months, peaking at 3675 microfilariae per milliliter (MF/mL) in Dog 1 (Yazoo-2013.1) at 411 days post infection and 5925 MF/mL in Dog 2 at 348 days post infection (Table 1). Microfilaremic blood from Dog 2 was used to feed mosquitoes to produce 32 L_3_ that were injected into a third dog. This infection was not challenged with ML treatment and is known as Yazoo-2013.2.

**Table 1:** Blood microfilaremia associated with experimental infections with two suspected ML-resistant isolates of *D. immitis*

Yazoo-2013.1			Metairie-2014	
Day post infection	Dog	MF/mL	Days post infection	MF/mL
212	Dog 1	1	183	0
	Dog 2	11		
246	Dog 1	75	215	50
	Dog 2	350		
286	Dog 1	550	245	1275
	Dog 2	975		
320	Dog 1	2400	280	9325
	Dog 2	4500		
348	Dog 1	2950	323	14,325
	Dog 2	5925		
381	Dog 1	2825	416	32,175
	Dog 2	3675		
411	Dog 1	3675	665	62,400
	Dog 2	3675		
446	Dog 1	2550	721	75,500
	Dog 2	4000		
			847	54,275

Only one dog was used in the isolation of Metairie-2014. Heartgard Plus was administered to the dogs at 30, 60, 90, 120, 150 and 180 days post infection

#### Metairie-2014

The Metairie-2014 isolate was obtained from a 13-year-old spayed female English retriever that resided in Metairie, Louisiana and traveled to Mississippi for hunting trips. The owner had a perfect purchase record of heartworm preventives. This dog became heartworm positive while on Trifexis® in March 2011 but was treated with melarsomine and doxycycline and was amicrofilaremic and antigen negative by the end of the year. Trifexis® was used from December 2013 through June 2014, when the dog tested heartworm antigen and MF positive again. Microfilaremic blood was sent to the University of Georgia and fed to mosquitoes, producing five L_3_ that were injected subcutaneously into a 2-year-old male purpose-bred mongrel dog. A second blood sample was sent 2 weeks later and fed to mosquitoes, yielding an additional 95 L_3_ that were injected into the same dog 9 days after the first five. Thirty days after all L_3_ had been introduced, Heartgard® Plus was administered according to weight. Drug treatment was repeated every 30 days for the 6 months following infection. Following the final drug administration, blood was drawn monthly to perform a thick smear to detect MF, which were first detected 7 months after infection. Heartworm antigen was detected by IDEXX SNAP® test in March 2015. Although no SNAP test was performed in February, due to the presence of MF it is reasonable to assume that the dog was also antigen positive. Microfilaremia rose steadily from days 215 to 847 post infection, peaking at 75,500 MF/mL of blood (Table 1).

### Isolation of MF from canine blood

Microfilariae were isolated from canine blood by two methods. The first is the standard Filariasis Research Reagent Resource Center (FR3) protocol first developed by Sawyer and Weinstein [19]. Canine blood in heparinized tubes was diluted 1:11 with 0.2% saponin in 0.85% NaCl to lyse red blood cells (RBC). Tubes were shaken several times to mix blood and saponin before being placed in a 38 °C water bath for approximately 30 min; tubes were shaken again halfway through incubation. Tubes were centrifuged for 15 min at 2500 RPM, and supernatant was discarded before washing MF twice with PBS. The PBS supernatant was removed and replaced with pre-warmed RPMI 1640 with L-glutamine containing 1% (*v*/v) penicillin/streptomycin (P/S). Microfilariae were placed in a 37 °C incubator with 5% CO_2_ for approximately 30 min before being counted.

The second method was developed by Franks and Stoll [20] and has been modified for use in our laboratory. Canine blood in heparinized tubes was centrifuged for 30 min at 2500 RPM before removing the top layer containing plasma and white blood cells. A 4:1 solution of 3.8% saline-citrate was added to bring the tube up to its original volume, and 1 mL of 15% (*w*/*v*) saponin was added for every 15 mL of blood. Tubes were shaken vigorously for 30 s before being centrifuged for 30 min at 2500 RPM. Supernatant was discarded and the pellet containing MF was washed twice with saline-citrate solution to remove any remaining RBC. Microfilariae were washed once with PBS before a final centrifugation for 4 min at 2500 RPM. The PBS supernatant was removed and replaced with pre-warmed RPMI 1640 with L-glutamine containing 1% P/S. Microfilariae were placed in a 37 °C incubator with 5% CO_2_ for approximately 30 min before being counted. Microfilariae were diluted or concentrated to 4 to 5 MF/μL for the Metairie and Yazoo 2013–2.1 strains and to 2 MF/μL for the Yazoo 2013–1 strain.

### Motility assays

The motility of the MF after 24 h in culture was assayed as described by Storey et al. [17], except that a heated stage top incubator (Tokai Hit model WSKM, Tokai Co. Ltd., Japan) was added to the microscope to maintain plates at 37 °C with 5% CO_2_ while they were being analyzed. Concentration-dependent inhibition of motility by ivermectin was assayed and analyzed as previously described [17]. In all cases, experiments were carried out in triplicate (*n* = 4–5).

## Results

### Motility of MF from ML-susceptible and resistant isolates and the effect of ivermectin

**Fig. 1:**
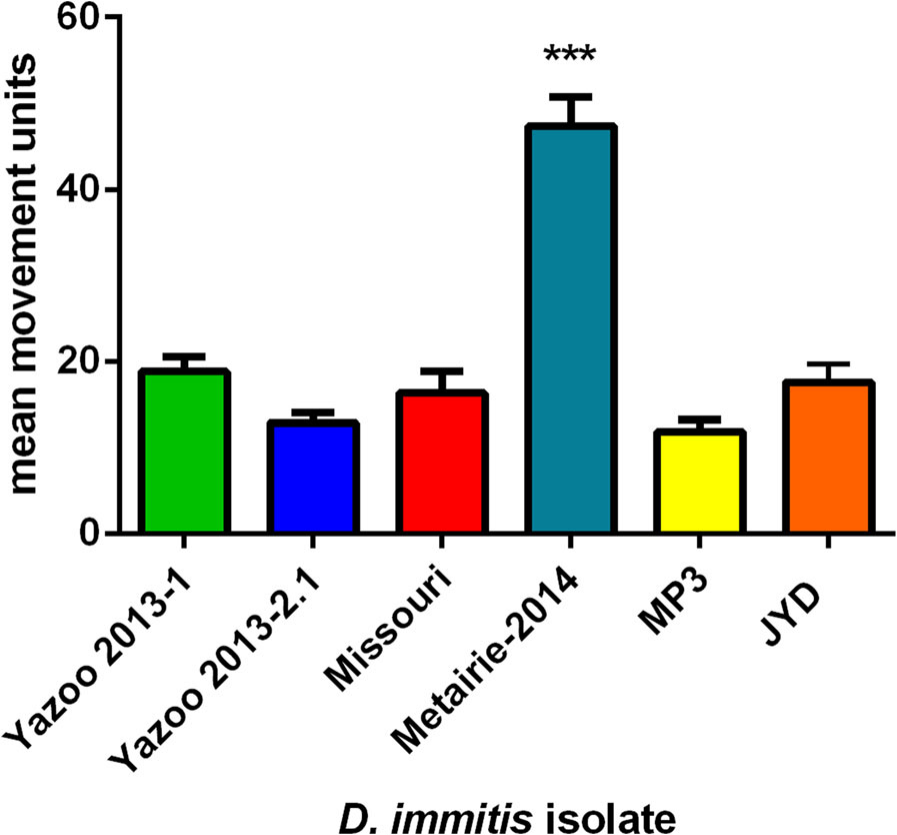
Baseline motility of *D. immitis* isolate microfilariae. Fifty microfilariae of each isolate were cultured for 24 h at 37 °C and 5% CO_2_. The larvae in individual wells were recorded for 30 s and the mean movement calculated using the WormAssay software [33]. Each experiment was performed in triplicate, *n* = 4

The motility of MF from these resistant isolates, plus the ML-resistant JYD and ‘less-susceptible’ MP3 [21, 22] was compared with that of the susceptible Missouri strain (Fig. 1) using the Worminator system [17]. There was no significant difference in the motility of Yazoo-2013.1 and Yazoo-2013.2 compared with Missouri, while Metairie was significantly more motile in the absence of any drug (*p* < 0.0001, two-way ANOVA, non-repeated measures) than all other strains.

**Fig. 2:**
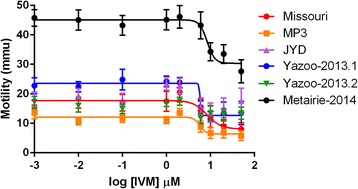
Dose-response curves for the effect of ivermectin on microfilariae motility

The effects of incubation with ivermectin on the motility of the isolated MF from the various isolates was measured (Fig. 2). Incubation of Missouri and MP3 MF in the presence of ivermectin for 24 h (1 nM to 50 μM) showed a concentration-dependent inhibition of motility with apparent IC_50_ values of 8.4 ± 1.6 μM and 5.8 ± 1.3 μM, respectively (Table 2 and Fig. 2). Despite similar baseline motility, differences between the passages of Yazoo-2013 in the presence of ivermectin were noted; the Yazoo-2013.1 MF showed a concentration-dependent reduction in motility (IC_50_ = 3.5 ± 1.6 μM) while Yazoo 2013.2 MF did not (Table 2 and Fig. 2). Metairie-2014 MF remained more motile than the other isolates in all drug concentrations, but also showed a concentration-dependent reduction in motility (IC_50_ = 8.1 ± 1.3 μM). The reduction in motility never exceeded 50% for any isolate, even at the maximum concentration we used (50 μM). The JYD MF showed no reduction in motility when incubated with ivermectin.

**Table 2:** Inhibition of microfilariae motility by ivermectin in vitro

Strain	IC_50_ μM IVM	Resistance status
Missouri	8.4 ± 1.6	susceptible
MP3	5.8 ± 1.3	‘less susceptible’
JYD	N/A	resistant
Yazoo 2013–1	3.5 ± 1.6	resistant
Yazoo 2013–2.1	N/A	resistant
Metairie	8.1 ± 1.3	resistant

Microfilariae of the isolates were isolated and incubated with various concentrations of ivermectin for 24 h at 37 °C in a 5% CO_2_ atmosphere. Motility was assessed using the ‘Worminator’ as described and the IC_50_ calculated using Prism 6

**Fig. 3:**
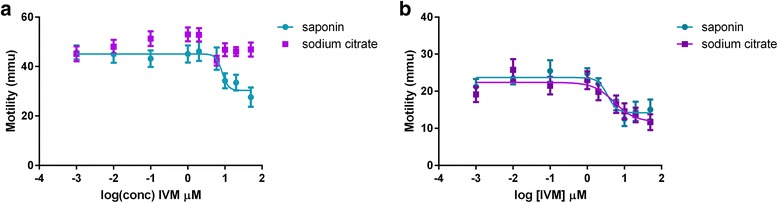
Effects of purification method on dose-response curves. A, Metairie-2014. B, Yazoo-2013.1

The inconsistencies observed with the results obtained with different resistant isolates, and between different passages of the same isolate, prompted the examination of the effects of the purification method on the motility and apparent drug sensitivity of the MF. Microfilariae from two resistant isolates were used, Yazoo-2013.1 and Metairie-2014, which exhibited a concentration-dependent inhibition of motility, and the effects of our standard ‘saponin’ purification procedure were compared with those of an alternative sodium citrate- based procedure. No effects of the purification procedure were observed on the motility of the MF in the absence of the drug. A marked difference, however, was seen in the effects of incubation with ivermectin on the Metairie-2014 MF; there was no inhibition of motility at any concentration (Fig. 3). The purification method had no effect, however, on the response of the Yazoo-2013.1 MF to the drug (Fig. 3).

## Discussion

Currently, the US Food and Drug Administration Center for Veterinary Medicine requires heartworm preventives to be 100% effective in preventing infection in order to be approved and licensed for use [7]. As described above, both Yazoo-2013 and Metairie-2014 isolates were able to establish patent infections in experimentally infected dogs despite six monthly drug challenges, unequivocally confirming their resistance to ML and that such parasites are in circulation in the United States [8, 9, 23]. Because considerable uncertainty remains about the current prevalence and the future impact of ML-resistant heartworms [2, 11, 12, 24], there is a major research need to develop a rapid, reproducible, and reasonably inexpensive assay to monitor the baseline prevalence and geographic spread of such biotypes. In vitro assays for anthelmintic resistance typically use a surrogate life-stage, rather than that which is the target of the drug treatments, and many assays, including some for ML resistance, use motility or an aspect of motility, such as migration, as a phenotypic read-out [25, 26]. We have previously described the use of a migration inhibition assay to measure the effects of MLs on *D. immitis* [13] and of high definition video recordings to do the same for MF [17]. Because the MF are much easier to obtain than L3, for future field application, we chose to examine the possibility of developing an assay for ML resistance using this life-stage.

The results were somewhat confusing. In the initial studies, MF from one resistant isolate, JYD, predictably showed no concentration-dependent inhibition of movement; however, another isolate, Metairie-2014, did. The results with the third resistant isolate, Yazoo-2013, were even more confusing, with the MF from passage 2 showing no ML-dependent inhibition, while those from passage 1 did. The IC_50_ values for Metairie-2014 and Yazoo-2013 were similar, in fact lower, than those for the two susceptible isolates, Missouri and MP3, indicating that there were no significant quantitative differences between the resistant and susceptible isolates in their response to ivermectin in this assay. All of the dose–response curves we obtained were similar to those previously reported [17] and confirm that much higher concentrations of drug are required to inhibit larval movement in vitro than are effective in vivo [27]. The difference in the numerical values of the IC_50_ reflects a difference in the calculation method. In our previous paper, the maximum value for inhibition was constrained to 100%, but because we never observed inhibition close to this in these experiments this constraint was not applied to the curves presented here. It is possible that using a drug other than ivermectin may work slightly better, as the use of eprinomectin seemed to work better than ivermectin in the L3 migration assay [13] and doramectin inhibits MF movement more robustly than ivermectin [17]. Cost considerations and solubility probably preclude the use of doramectin in a routine assay, however, and the discrepancy in concentration required for effects in vitro and in vivo remains. Eprinomectin also fails to distinguish between susceptible and resistant L3 in the LMIA [14].

An additional confounding factor was revealed by varying the purification method use to obtain the MF. Our standard protocol is based on that described by Sawyer and Weinstein [19]; when we compared the results with MF purified with this method with those purified by the method of Franks and Stoll [20], the apparent phenotype of the Metairie-2014 isolate changed from ‘susceptible’ to ‘resistant.’ We do not know the reason for this change, but one speculation is that the MF isolated via the ‘saponin’ method [19] may be slightly more damaged than those isolated via the ‘citrate’ method [20], and this may allow the drug to have a greater effect. The fact that the purification method only affected Metairie-2014 but not Yazoo-2013.2 might point to differences in the physical robustness of the two isolates, but this possibility needs further investigation.

Overall, our data suggest that simple motility measurements on MF are unlikely to form the basis of a robust assay for ML resistance in heartworm. This may not be surprising, as the glutamate-gated chloride channels that are the molecular targets of the drugs [28–30] have multiple roles in many other nematode species and do not appear to be expressed in the motor nervous system of MF [31]. Taken together with evidence suggesting that the anti-MF effect of the MLs may require a contribution from the host immune system [27, 32], our results indicate that motility of MF is not a reliable phenotype for detecting resistance in *D. immitis*. This likely reflects the fact that the drug concentrations experienced by the parasites in the host are several orders of magnitude lower than those required to inhibit motility in vitro. Thus, it would seem that inhibition of motility is not a mechanism of action for the ML drugs against *D. immitis* and thus is unlikely to be a mechanism of resistance. Data from this study indicate that for the moment an in vivo assay for resistance such as the microfilarial suppression test [12] may be the most useful means of determining whether resistance is present in a suspect case.

## Abbreviations

IVM: Ivermectin
MF: Microfilariae
ML: Macrocyclic lactone
Mmu: Mean movement units
